# Let Us Know Transfusion Triggers for Prophylactic Use of Platelet Concentrate—Analysis of Compliance with Recent Transfusion Guidelines in a Large Academic Medical Center

**DOI:** 10.3390/jcm12185885

**Published:** 2023-09-10

**Authors:** Piotr F. Czempik, Jan Herzyk, Dawid Wilczek

**Affiliations:** 1 Department of Anesthesiology and Intensive Care, Faculty of Medical Sciences in Katowice, Medical University of Silesia, 40-752 Katowice, Poland; 2 Transfusion Committee, University Clinical Center of Medical University of Silesia in Katowice, 40-752 Katowice, Poland; 3 Students’ Scientific Society, Department of Anesthesiology and Intensive Care, Faculty of Medical Sciences in Katowice, Medical University of Silesia, 40-752 Katowice, Poland

**Keywords:** decision making, guideline, platelet concentrate, thrombocytopenia, transfusion

## Abstract

Platelet concentrate (PC) is a blood component that is used to prevent or manage bleeding associated with thrombocytopenia or impaired platelet function. The aim of our study was to assess the compliance of ordering physicians with the most recent PC transfusion guidelines in our academic medical center. All PC transfusions performed between January 2019 and December 2022 were analyzed. The appropriateness of PC transfusions was assessed based on the most recent PC transfusion guidelines. During 2019–2022, there were 362 (0.2%) PC recipients out of 161,762 hospitalized patients. There were 971 PCs transfused during the analyzed period. Inappropriate transfusions accounted for 53.3% of cases, and most of them were given prophylactically (80.2%). Compliance with platelet transfusion guidelines varied among departments. The overall percentage of inappropriately transfused PC ranged from 50.7% to 60.8% in successive years. Educational activities should target clinicians performing procedures associated with high rates of inappropriate PC transfusions. Implementing clinical decision support systems can help reduce unnecessary PC transfusions and associated costs. The majority of inappropriate PC transfusions in our medical center were given as prophylaxis against bleeding. Prescribers should be educated about evidence-based transfusion triggers for the prophylactic use of PC in various clinical scenarios.

## 1. Introduction

Transfusion guidelines for different blood components are periodically updated [1,2]. The rationale for new iterations of transfusion guidelines is accumulating evidence. Transfusion guidelines aim to improve the risk-benefit balance of blood component use. Clinicians are advised to follow these guidelines. However, dissemination and implementation of clinical guidelines vary across institutions and even between individual prescribers [3].

Blood components have the potential to save lives in the event of bleeding. They are also commonly transfused as prophylaxis for spontaneous bleeding or associated with invasive procedures. Platelet concentrate (PC) is a blood component that is used to prevent or manage bleeding associated with thrombocytopenia or impaired platelet function [4]. Although PC has been traditionally used to stop bleeding, its hemostatic effect has not been well researched, and new models for hemostatic efficacy testing have been proposed [5]. On the other hand, transfusion of PC, like other blood components, may lead to infectious and, more commonly, non-infectious complications [6]. The non-infectious acute transfusion reactions include febrile non-hemolytic transfusion reaction (FNHTR) (1 per 14 transfusions), allergic transfusion reaction (1 per 50 transfusions), anaphylaxis, acute hemolytic transfusion reaction, transfusion-associated circulatory overload (TACO), transfusion-related acute lung injury (TRALI) (1 per 138,000), transfusion-associated graft vs. host disease (TA-GvHD) (1–10 per 1000), with an estimated mortality reaching up to 100%, and alloimmunization [7,8,9,10,11,12]. The less researched effects of PC are the increased risk of thrombosis and immunomodulation (transfusion-related immunomodulation, TRIM), as platelets have both hemostatic and immunologic functions. Pre-procedure PC transfusion has been linked with thrombosis and increased 30-day mortality [13]. Transfusion-related immunomodulation is due to the modified response of T-helper (1 and 2) cells, which predisposes to infection and delays recovery after surgery [14]. The most common infectious complication of PC transfusion is bacterial infection (contamination of PC) (1 per 75,000). Due to numerous possible complications, if PC is used outside the recommended indications, patients are put at risk. Poor implementation of blood component transfusion guidelines leads to over-transfusion, as the authors showed in the previous publication regarding red blood cells (RBC) [15]. Over-transfusion of PC also decreases the availability of this scarce resource, which is particularly important during pandemics when recruitment of donors is compromised, an example being the novel coronavirus disease (COVID-19) pandemic [16]. The most recent PC transfusion guidelines were published in 2015 by the Association for the Advancement of Blood and Biotherapies and in 2017 by the British Society of Hematology (BSH) [2,17].

Taking into account the important matter of appropriate use of PC in clinical medicine, the aim of our study was to assess the compliance of ordering physicians with the most recent PC transfusion guideline in our medical center [2].

## 2. Materials and Methods

All PC transfusions performed in our medical center between January 2019 and December 2022 were analyzed. All data were retrieved from the electronic health records (AMMS, Asseco Medical Solutions, Rzeszów, Poland). Apart from basic demographic data (age, sex), the following clinical data were retrieved: hospital department where PC transfusion was performed, indication for PC transfusion, number of platelets (PLT) and hemoglobin (Hb) concentrations up to 24 h before and after PC transfusion, and number and type of PC transfused. The transfusion rate (TR), i.e., percent of patients transfused, as well as the number of PCs per hospitalized patient, were calculated. The transfusion index (TI), i.e., mean number of PC transfused in a recipient, and transfusion incidence, i.e., number of PC transfusions in a population during a time period (expressed as *n* per 1000 person-days), were also calculated. These transfusion metrics were calculated for the general population and patients hospitalized in various hospital departments, as well as for the entire analyzed period and the successive years within. We performed an additional analysis of transfusion reactions and adverse transfusion events associated with PC transfusion.

Platelet concentrates are derived from whole blood from multiple donors or collected by apheresis from a single donor. Platelet concentrates can undergo some modifications in order to reduce the risk of adverse events: pathogen reduction (bacterial contamination), leucoreduction (FNHTR, risk of CMV transmission, HLA alloimmunization), irradiation (TA-GvHD), washing (allergy, alloimmunization), or volume reduction (allergy, alloimmunization, TACO) [18].

The appropriateness of PC transfusions was assessed based on the most recent PC transfusion guidelines [2]. These guidelines were published prior to the analyzed period. Apart from assessing compliance with PC transfusion guidelines in individual departments, the implementation of guidelines over time was also assessed. The summary of therapeutic and prophylactic clinical situations where PC transfusion was considered appropriate according to these guidelines is presented in Table 1.

The severity of bleeding was based on the modified World Health Organization (WHO) bleeding score [19]. Reversible bone marrow failure is where recovery of PLT is anticipated. Hypoproliferative thrombocytopenia denotes chronic bone marrow failure where recovery is not anticipated. Although the BSH guidelines do not recommend or suggest giving PC transfusion routinely prior to bone marrow aspirate or trephine biopsy, peripherally inserted central catheters (PICC), traction removal of tunneled central venous catheters (CVC), or cataract surgery, we decided that PC transfusion at PLT < 10 × 10^9^/L was warranted in most of the cases as this a PLT threshold is used to prevent spontaneous bleeding. Although the BSH guidelines recommend a PLT count of 80 × 10^9^/L when spinal anesthesia is performed, they also mention that a PLT count of 40 × 10^9^/L threshold would be more logical.

Additional risk factors for bleeding denote risk factors like infection, fever, other hemostatic derangements, an abrupt decrease in platelet number, use of medications increasing the risk of bleeding, anemia, and uremia. The common medications that increase the risk of bleeding are as follows: acetylsalicylic acid, clopidogrel, ticagrelor, non-steroidal anti-inflammatory drugs, selective serotonin reuptake inhibitors, and valproic acid. The appropriateness of PC transfusions was assessed for the whole analyzed period as well as for successive years during the period in order to analyze if implementation of guidelines improved over time.

The statistical analysis was performed with MedCalc version 18 statistical software (MedCalc Software, Ostend, Belgium). Continuous variables were reported as medians and interquartile ranges (IQR) due to their non-normal distribution. We confirmed the distribution using the Shapiro-Wilk test. Categorical variables were presented as numbers and percentages.

Due to the retrospective nature of the study, the Bioethics Committee of the Medical University of Silesia, Katowice, Poland, waived the requirement for this analysis to be assessed by the Committee (decision number: PCN/CBN/0022/KB/292/21).

## 3. Results

In the analyzed period, there were 362 PC recipients and a total of 161,762 hospitalized patients. The median age of PC recipients was 62 (IQR 46.0–70.0) years, and there were 165 (45.6%) men and 197 (54.4%) women with median ages of 64 (IQR 49.7–71.0) and 61 (IQR 43.0–70.0) years, respectively. The metrics of PC transfusions during the analyzed period are presented in Table 2.

The TR has stayed stable over the last 2 years at 0.23%. The TI stayed the same over the entire analyzed period. The transfusion incidence varied from 1.54 to 2.07 transfusions per 1000 patient-days.

There were 971 PCs transfused during the analyzed period. The numbers of different types of PC transfused were as follows: apheresis irradiated (447, 46.0%), pooled (216, 22.2%), apheresis (215, 22.1%), pooled irradiated (81, 8.3%), pooled thawed (5, 0.5%), apheresis thawed washed irradiated (3, 0.3%), apheresis reconstituted (2, 0.2%), pooled inactivated (riboflavin) (2, 0.2%).

The metrics of platelet concentrate transfusions in different hospital departments in the analyzed period are presented in Table 3.

The overall TR, TI, and transfusion incidence were 0.22%, 2.0 (IQR 1.0–3.0) PCs per recipient, and 1.81 PCs per 1000 patient-days, respectively. The hospital department where TR and transfusion incidence were significantly higher than in other departments was the intensive care unit (ICU)—5.07% and 18.59 PCs per 1000 patient-days, respectively. There were no transfusion reactions (mild to life-threatening) or adverse transfusion events associated with PC transfusion in the analyzed period.

Overall, the median PLT before and after PC transfusion was 28 (IQR 16.0–50.5) and 52 (IQR 31.0–82.5) × 10^9^/L (*p* < 0.01), respectively. The median Hb concentration before and after PC transfusion was 91 (IQR 77–109) and 87 (IQR 77–102) g/L (*p* = 0.03), respectively. Overall, there were 434 (44.7%) appropriate and 537 (53.3%) inappropriate PC transfusions (Table 4).

In general, 80.2% of inappropriate PC transfusions were given as prophylaxis and 19.8% as treatment for bleeding. As many as 335 (62.5%) inappropriate PC transfusions were given in 2 prophylactic and 1 therapeutic clinical scenarios: bone marrow dysfunction or critical illness with additional bleeding risk factors (30.2%), hypoproliferative thrombocytopenia with additional bleeding risk factors (18.6%), and mild bleeding (13.6%), respectively. The number of inappropriate PC transfusions before endoscopic procedures with biopsy was 7 (33.3%), whereas before endoscopic procedures without biopsy was 41 (87.2%).

Compliance with platelet transfusion guidelines differed between hospital departments. More than 50% of PC transfusions were inappropriate in the following hospital departments: gynecology, gastrointestinal surgery, oncological surgery, clinical pharmacology, neurosurgery, gastroenterology, and the stroke unit. Taking into account the number of all inappropriate PC transfusions, the highest percentage was transfused in the following hospital departments: ICU (19.4%), Autoimmune (16.4%), Gynecology (15.3%), Gastroenterology (14.4%), and Clinical Pharmacology (11.5%), for a total of 77% of inappropriate PC transfusions shown in Table 5.

The percentage of inappropriately transfused PCs (all clinical scenarios) for the successive years were as follows: 60.7, 50.9, 50.7, and 60.8%.

## 4. Discussion

Numerous studies point out that PC or RBC transfusions are performed outside of the recommendations provided in guidelines [15,20]. In our study, we showed that approximately 56% of all PC transfusions were not compliant with the most recent PC transfusion guidelines [2]. This percentage is higher than published by authors who showed that 41.5% of adult PC orders were deemed inappropriate [21]. Reported reasons for overriding blood transfusion guidelines were diverse, including acute bleeding (>34%), protocolized behaviors on specialty services (up to 26%), “symptomatic” anemia (11–12%), anticipated surgical/procedural intervention (10–15%), and imminent hospital discharge (2–5%) [22]. In our case, the reasons for the higher than reported percentage of inappropriate PC transfusions could be the lack of a hematology department in our medical center and the fact that patients with hematological diagnoses are hospitalized in internal medicine departments, where compliance with PC transfusion guidelines is generally lower. Taking into account the number of PC recipients and high PC transfusion incidence in these two departments (Table 3), this could affect the results obtained.

In the study by Hill-Strathy et al., the most common clinical scenario where PC transfusion was considered inappropriate (53% of inappropriate orders in adults) was prophylaxis in the absence of bleeding or before the planned procedure in patients with hypo-proliferative thrombocytopenia and a platelet count over 10 × 10^9^/L [21]. The percentage of inappropriate PC transfusions in our study was much higher than in the population of hematology patients, in whom only 28% of transfusions were performed outside of guidelines [23]. However, such a low percentage of inappropriate PC transfusions could be due to the relatively long time passed between the publication of the PC transfusion guidelines on which the appropriateness was based and the analyzed period (approx. 9 years) and perhaps the medical specialty of the ordering clinicians (hematologists). In our study, prophylaxis in patients with hypo-proliferative thrombocytopenia with additional bleeding risk factors constituted 18.6% of all inappropriately transfused PCs (transfusions performed in patients with pre-transfusion PLT ≥ 20 × 10^9^/L). In our study, 80.2% of all inappropriate PC transfusions were given as prophylaxis and only 19.8% as treatment for bleeding. In the study by Etchells et al., 22% of adult PC orders were deemed inappropriate, and the appropriateness of prophylactic transfusions for non-bleeding patients compared to therapeutic transfusions for bleeding patients was 85% vs. 73% [24].

We decided to evaluate PC transfusion practice in different clinical situations in order to formulate suggestions for improvement. By showing a high percentage of inappropriate PC transfusions as pre-procedure prophylaxis, educational activities could be aimed at the clinicians performing these procedures. The highest percentage of inappropriate PC transfusions were performed as prophylaxis before lumbar puncture, bone marrow biopsy, and endoscopic procedures without biopsy. In clinical scenarios like paracentesis, insertion/removal of an epidural catheter, percutaneous liver biopsy, multiple trauma, and immune thrombocytopenia, all PC transfusions were deemed appropriate. It seems that in rarely performed procedures or clinical scenarios, the approach of clinicians towards PC transfusion is more conservative.

There is no evidence that prophylactic PC transfusion before surgery reduces postoperative bleeding or all-cause mortality [2]. There is also no evidence that a prophylactic PC transfusion before chemotherapy and stem cell transplantation prevents bleeding in hematological patients [23]. Moreover, there is no evidence that PC dose affects the incidence of WHO grade 4 bleeding [23]. Nevertheless, prophylactic PC transfusions are more effective than platelet-poor plasma in preventing bleeding [23]. Taking these into account, the implementation of PC transfusion guidelines is an important issue for reaching the best possible risk-benefit balance.

In our study, the highest number of inappropriate PC transfusions (nearly 80%) were carried out in 5 hospital departments: ICU, autoimmune, clinical pharmacology, gynecology, and gastroenterology. On the other hand, Etchells et al. showed that the highest percentage of inappropriate PC transfusions were performed by general surgery services [24]. This result was different from our study, probably due to differences in local services or the characteristics of hospitalized patients. Educational activities should particularly be aimed at prescribers from departments where audits showed a high percentage of PC over-transfusion. Mandatory training in transfusion medicine contributes to increased adherence to blood transfusion recommendations at a relatively low cost [25].

Implementation of clinical decision support (CDS) systems may reduce unnecessary PC transfusions [3]. It has been described in 1998 by Hunt et al. as “any software designed to directly aid in clinical decision-making in which characteristics of individual patients are matched to a computerized knowledge base for the purpose of generating patient-specific assessments or recommendations that are then presented to clinicians for consideration” [26]. These systems usually lead to an increased percentage of single-unit transfusions, which translates into a decrease in the number of blood components used while the number of orders remains unchanged [27,28]. It is also correlated with the reduction of costs associated with these procedures [29]. Implementing this method can lead to an immediate reduction of unnecessary blood components; however, the physician should be mindful of the potential for off-label use [30]. Even in the case of massive transfusions, CDS can contribute to improved patient safety, patient outcomes, and blood utilization [31]. These systems are enhancing adherence to transfusion medicine evidence [32]. This intervention can easily be replicated in other hospitals by using the software available there for the day-to-day management of the ward [33].

The advantages of using specific platelet thresholds for transfusion are still not clarified. Prophylactic pre-procedure PC transfusions are associated with an increased rate of 7-day post-procedure thrombosis (5.0%) and 30-day mortality (16.0%) [13]. In sepsis patients with thrombocytopenia, PC transfusion is also associated with higher short-term (28 days) and long-term (90 days) mortality [34]. In contrast to this, platelet transfusion in sepsis patients with higher platelet counts was associated with decreased mortality compared to patients with lower baseline platelet counts [35]. In our study, we did not perform follow-up on PC recipients.

We did not report in our study any transfusion reactions or adverse transfusion events associated with PC transfusion. This was quite remarkable to us because even mild allergic reactions were not reported. In our opinion, this could be caused by the underreporting of mild transfusion reactions by attending physicians.

Due to its retrospective nature, the study is not devoid of limitations. Some clinical information might have been lost due to not being recorded in the hospital information technology system. Therefore, despite careful analysis of daily patient hospitalization records drafted by attending physicians, with a special emphasis on descriptions related to PC transfusions, certain transfusions could have been inappropriately qualified as inappropriate.

## 5. Conclusions

The majority of PC transfusions in our large academic medical center were given as prophylaxis against bleeding. Although the incidence of PC transfusions in our study was generally low, more than half of all PC transfusions were performed outside of recommended indications. Inappropriate PC transfusions were most frequently performed for prophylactic purposes. Prescribers should be educated about evidence-based transfusion triggers for the prophylactic use of PC in various clinical scenarios.

## Figures and Tables

**Table 1:** Summary of appropriate platelet concentrate transfusion clinical situations.

Platelets	Clinical Situation
	Therapeutic Use
**Unspecified**	Glanzmann thrombasthenia—after administration of rVIIa ^1^
**Unspecified**	Other platelet function disorders—after administration of TXA ^2^ and desmopressin
**Unspecified**	Post-transfusion purpura—after administration of intravenous immunoglobulins
**Unspecified**	Immune thrombocytopenia—intravenous immunoglobulins may be considered, PC ^3^ given only before procedures
**<30 ×** **10^9^/L**	Mild bleeding (WHO ^4^ 0–2) [19]
**<50 ×** **10^9^/L**	Severe bleeding (WHO 3–4) [19]
**<100 ×** **10^9^/L**	Multiple trauma, traumatic brain injury, ocular trauma, spontaneous cerebral bleeding
	**Prophylactic use**
**<10 ×** **10^9^/L**	reversible bone marrow dysfunction (no additional risk factors), hypoproliferative thrombocytopenia, bone marrow biopsy, cataract surgery, PICC ^5^ insertion, CVC ^6^ removal (no additional risk factors)
**10–20 ×** **10^9^/L**	Reversible bone marrow dysfunction/hypoproliferative thrombocytopenia/critical illness (additional risk factors)
**<20 ×** **10^9^/L**	endoscopic procedures without biopsy, angiography, minor surgical procedures, CVC insertion by an experienced operator under ultrasound guidance, bronchoscopy, paracentesis, thoracentesis
**<40 ×** **10^9^/L**	Lumbar puncture, spinal anesthesia
**<50 ×** **10^9^/L**	Endoscopic procedures with biopsy, percutaneous liver biopsy, major surgical procedures
**<80 ×** **10^9^/L**	Insertion/removal of epidural catheter
**<100 x** **10^9^/L**	Neurosurgical and ophthalmic (posterior chamber) procedures

^1^ rVIIa—recombinant activated factor VII; ^2^ TXA—tranexamic acid; ^3^ PC—platelet concentrate; ^4^ WHO—World Health Organization; ^5^ PICC—peripherally inserted central catheter; ^6^ CVC—central venous catheter.

**Table 2:** The metrics of platelet concentrate transfusions during the analyzed period.

Year	Recipients [*n*]	All Patients [*n*]	TR ^1^ [%]	PC ^2^ per 1000 pts [*n*]	TI ^3^ [*n*, IQR ^4^]	Transfusion Incidence [*n*/1000 Patient-Days]
2019	80	43,053	0.19	5.20	2 (1–3)	1.54
2020	88	34,479	0.26	6.15	2 (1–2)	1.84
2021	95	40,921	0.23	6.84	2 (1–3)	2.07
2022	99	43,309	0.23	5.89	2 (1–3)	1.82
2019–2022	362	161,762	0.22	6.00	2 (1–3)	1.81

^1^ TR—transfusion rate; ^2^ PC—platelet concentrate; ^3^ TI—transfusion index; ^4^ IQR—Interquartile Range.

**Table 3:** The metrics of platelet concentrate transfusions in different hospital departments in the analyzed period.

Hospital Department	Recipients [*n*]	All Patients [*n*]	TR ^1^ [%]	PC ^2^ per 1000 pts [*n*]	TI ^3^ [*n*, IQR ^4^]	Transfusion Incidence [*n*/1000 Patient-Days]
Intensive Care Unit	83	1638	5.07	131.87	2.0 (1.0–3.0)	18.59
Autoimmune	38	3907	0.97	46.58	3.0 (2.0–5.0)	7.26
Clin. Pharmacology	34	2794	1.22	34.72	2.0 (1.0–4.0)	4.25
Gastroenterology	63	13,353	0.47	10.56	1.0 (1.0–2.0)	2.38
Neurosurgery	36	6260	0.58	12.14	2.0 (1.0–2.0)	1.94
Gynecology	39	16,087	0.24	6.40	2.0 (2.0–2.0)	1.61
Radiotherapy	6	3399	0.18	7.94	4.0 (2.0–5.0)	1.59
Oncology 2	12	15,950	0.08	1.94	2.0 (1.0–4.0)	1.27
Gastrointestinal Surg.	24	5222	0.46	7.66	1.0 (1.0–2.0)	1.17
Oncology 1	8	18,816	0.04	1.12	2.5 (1.0–4.0)	0.79
Neurology	8	7007	0.11	2.85	2.0 (1.5–2.5)	0.72
Stroke Unit	7	2267	0.31	5.73	2.0 (2.0–2.0)	0.57
Oncological Surgery	3	3514	0.09	0.85	1.0	0.28
Ophthalmology	1	43,302	0.00	0.02	1.0	0.01
Rehabilitation	0	686	-	-	-	-
Endocrine Gynecology	0	4251	-	-	-	-
Alergology	0	5847	-	-	-	-
Endocrinology	0	7462	-	-	-	-
Total	362	compliance	0.22	6.00	2.0 (1.0–3.0)	1.81

^1^ TR—transfusion rate; ^2^ PC—platelet concentrate; ^3^ TI—transfusion index; ^4^ IQR—Interquartile Range.

**Table 4:** The appropriateness of platelet concentrate transfusions in different clinical situations.

Clinical Situation	Platelet Concentrate Transfusions			
	All [*n*, %]	Appropriate [*n*, %]	Inappropriate [*n*, %]	Percentage of All Inappropriate [%]
Marrow dysfunction or critical illness (RFs ^1^)	292 (30.1)	130 (44.5)	162 (55.5)	30.2
Hypoproliferative thrombocytopenia (RFs)	154 (15.9)	54 (35.1)	100 (64.9)	18.6
Mild bleeding	172 (17.7)	99 (57.6)	73 (42.4)	13.6
Major surgical procedures	73 (7.5)	30 (41.1)	43 (58.9)	8.0
Endoscopic procedures without biopsy	47 (4.8)	6 (12.8)	41 (87.2)	7.6
Neurosurgical/ophthalmic procedures	64 (6.6)	28 (43.7)	36 (56.3)	6.7
Severe bleeding	66 (6.8)	37 (56.1)	29 (43.9)	5.4
Minor surgical procedures	20 (2.1)	5(25.0)	15 (75.0)	2.8
Spinal anesthesia	18 (1.9)	4 (22.2)	14 (77.8)	2.6
Spontaneous cerebral bleeding	25 (2.6)	18 (72.0)	7 (28.0)	1.3
Endoscopic procedures with biopsy	21 (2.2)	14 (66.7)	7 (33.3)	1.3
Central venous catheter insertion	8 (0.8)	1 (12.5)	7 (87.5)	1.3
Lumbar puncture	2 (0.2)	0 (0.0)	2 (100.0)	0.4
Bone marrow biopsy	1 (0.1)	0 (0.0)	1 (100.0)	0.2
Immune thrombocytopenia	4 (0.4)	4 (100.0)	0 (0.0)	0.0
Multiple trauma	1 (0.1)	1 (100.0)	0 (0.0)	0.0
Percutaneous liver biopsy	1 (0.4)	1 (100.0)	0 (0.0)	0.0
Insertion/removal epidural catheter	1 (0.1)	1 (100.0)	0 (0.0)	0.0
Paracentesis	1 (0.1)	1 (100.0)	0 (0.0)	0.0
Bronchoscopy	0 (0.0)	0 (0.0)	0 (0.0)	0.0
Thoracentesis	0 (0.0)	0 (0.0)	0 (0.0)	0.0
All	971 (100)	434 (44.7)	537 (53.3)	100

^1^ RFs—risk factors for bleeding.

**Table 5:** The appropriateness of platelet concentrate transfusions in different hospital departments during the study period.

Hospital Department	Platelet Concentrate Transfusions		
	Appropriate [*n*, %]	Inappropriate [*n*, %]	Percentage of All Inappropriate [%]
Intensive Care Unit	111 (51.4)	105 (48.6)	19.4
Autoimmune	93 (51.1)	89 (48.9)	16.4
Gynecology	20 (19.4)	83 (80.6)	15.3
Gastroenterology	63 (44.7)	78 (55.3)	14.4
Clinical Pharmacology	35 (36.1)	62 (63.9)	11.5
Neurosurgery	31 (40.8)	45 (59.2)	8.3
Gastrointestinal Surgery	12 (30.0)	28 (70.0)	5.2
Radiotherapy	15 (55.6)	12 (44.4)	2.2
Oncology 2	20 (64.5)	11 (35.5)	2.0
Neurology	10 (50.0)	10 (50.0)	1.8
Oncology 1	13 (61.9)	8 (38.1)	1.5
Stroke Unit	6 (46.2)	7(53.8)	1.3
Oncological Surgery	1 (33.3)	2 (66.7)	0.4
Ophthalmology	0 (0.0)	1 (100)	0.2
Rehabilitation	0 (0.0)	0 (0.0)	0.0
Endocrine Gynecology	0 (0.0)	0 (0.0)	0.0
Alergology	0 (0.0)	0 (0.0)	0.0
Endocrinology	0 (0.0)	0 (0.0)	0.0
Total	434 (44.7)	537 (53.3)	100

## Data Availability

The data presented in this study are available upon request from the corresponding authors.
